# Biological Behavior of Xenogenic Scaffolds in Alcohol-Induced Rats: Histomorphometric and Picrosirius Red Staining Analysis

**DOI:** 10.3390/polym14030584

**Published:** 2022-01-31

**Authors:** Dayane Maria Braz Nogueira, André Luiz de Faria Figadoli, Patrícia Lopes Alcantara, Karina Torres Pomini, Iris Jasmin Santos German, Carlos Henrique Bertoni Reis, Geraldo Marco Rosa Júnior, Marcelie Priscila de Oliveira Rosso, Paulo Sérgio da Silva Santos, Mariana Schutzer Ragghianti Zangrando, Eliana de Souza Bastos Mazuqueli Pereira, Miguel Ângelo de Marchi, Beatriz Flavia de Moraes Trazzi, Jéssica de Oliveira Rossi, Samira Salmeron, Cláudio Maldonado Pastori, Daniela Vieira Buchaim, Rogerio Leone Buchaim

**Affiliations:** 1 Department of Prosthodontics and Periodontics, Bauru School of Dentistry (FOB/USP), University of São Paulo, Bauru 17012-901, Brazil; dayanenogueira@usp.br (D.M.B.N.); mariana@fob.usp.br (M.S.R.Z.); s.salmeron@usp.br (S.S.); 2 Department of Biological Sciences, Bauru School of Dentistry (FOB/USP), University of São Paulo, Bauru 17012-901, Brazil; afigadoli@gmail.com (A.L.d.F.F.); karinatp@usp.br (K.T.P.); irisgerman@usp.br (I.J.S.G.); dr.carloshenriquereis@usp.br (C.H.B.R.); marcelierosso@usp.br (M.P.d.O.R.); 3 Department of Surgery, Stomatology, Pathology and Radiology, Bauru School of Dentistry, University of São Paulo, Bauru 17012-901, Brazil; patricia.alcantara@usp.br (P.L.A.); paulosss@fob.usp.br (P.S.d.S.S.); 4 Postgraduate Program in Structural and Functional Interactions in Rehabilitation, Postgraduate Department, University of Marilia (UNIMAR), Marília 17525-902, Brazil; elianabastos@unimar.br (E.d.S.B.M.P.); danibuchaim@alumni.usp.br (D.V.B.); 5 Technical Board, UNIMAR Beneficent Hospital (HBU), University of Marilia (UNIMAR), Marília 17525-160, Brazil; 6 Anatomy Discipline, School of Dentistry, Health Sciences Center, Sacred Heart University Center (UNISAGRADO), Bauru 17011-160, Brazil; geraldo.junior@unisagrado.edu.br; 7 Coordination of the Medical School, Medical School, University Center of Adamantina (UniFAI), Adamantina 17800-000, Brazil; coordmedicina@fai.com.br; 8 Coordination of the Dentistry School, Dentistry School, University of Marilia (UNIMAR), Marília 17525-902, Brazil; flavia.odonto@unimar.br; 9 Graduate Program in Anatomy of Domestic and Wild Animals, Faculty of Veterinary Medicine and Animal Science, University of São Paulo (FMVZ/USP), São Paulo 05508-270, Brazil; jessicagoncalves@alumni.usp.br; 10 Dentistry School, University Center of Adamantina (UniFAI), Adamantina 17800-000, Brazil; claudiomaldonado@fai.com.br; 11 Teaching and Research Coordination, Medical School, University Center of Adamantina (UniFAI), Adamantina 17800-000, Brazil

**Keywords:** bone regeneration, alcohol intake, scaffolds, bone repair, tibia, xenograft, biomaterials

## Abstract

In this experimental protocol, the objective was to evaluate the biological behavior of two xenogenic scaffolds in alcohol-induced rats through histomorphometric and Picrosirius Red staining analysis of non-critical defects in the tibia of rats submitted or not to alcohol ingestion at 25% *v*/*v*. Eighty male rats were randomly divided into four groups (*n* = 20 each): CG/B (water diet + Bio-Oss^®^ graft, Geistlich Pharma AG, Wolhusen, Switzerland), CG/O (water diet + OrthoGen^®^ graft, Baumer, Mogi Mirim, Brazil), AG/B (25% *v*/*v* alcohol diet + Bio-Oss^®^ graft), and AG/O (25% *v*/*v* alcohol diet + OrthoGen^®^ graft). After 90 days of liquid diet, the rats were surgically obtained, with a defect in the tibia proximal epiphysis; filled in according to their respective groups; and euthanized at 10, 20, 40 and 60 days. In two initial periods (10 and 20 days), all groups presented biomaterial particles surrounded by disorganized collagen fibrils. Alcoholic animals (AG/B and AG/O) presented, in the cortical and medullary regions, a reactive tissue with inflammatory infiltrate. In 60 days, in the superficial area of the surgical cavities, particles of biomaterials were observed in all groups, with new compact bone tissue around them, without complete closure of the lesion, except in non-alcoholic animals treated with Bio-Oss^®^ xenograft (CG/B), where the new cortical interconnected the edges of the defect. Birefringence transition was observed in the histochemical analysis of collagen fibers by Picrosirius Red, in which all groups in periods of 10 and 20 days showed red-orange birefringence, and from 40 days onwards greenish-yellow birefringence, which demonstrates the characteristic transition from the formation of thin and disorganized collagen fibers initially to more organized and thicker later. In histomorphometric analysis, at 60 days, CG/B had the highest volume density of new bone (32.9 ± 1.15) and AG/O the lowest volume density of new bone (15.32 ± 1.71). It can be concluded that the bone neoformation occurred in the defects that received the two biomaterials, in all periods, but the Bio-Oss^®^ was superior in the results, with its groups CG/B and AG/B displaying greater bone formation (32.9 ± 1.15 and 22.74 ± 1.15, respectively) compared to the OrthoGen^®^ CG/O and AG/O groups (20.66 ± 2.12 and 15.32 ± 1.71, respectively), and that the alcoholic diet interfered negatively in the repair process and in the percentage of new bone formed.

## 1. Introduction

Alcohol consumption is often accepted and socially encouraged, so its consumption has become excessive, causing serious damage and harm to the health of its consumers [1,2,3,4,5]. The term alcoholism is characterized by the abusive and compulsive ingestion of alcoholic beverages, being considered a chronic disease, affecting millions of people in the world, considered highly toxic to vital organs and tissues [6,7]. Chronic consumption can cause disturbances and damage such as organic and psychological disorders, central nervous system, muscle system, bone tissue, liver, cardiovascular diseases and (especially) socioeconomic damage [8].

One of the tissues harmed by chronic alcohol consumption is the bone tissue [9]. Alcohol acts on bone change, changing the process of bone resorption and formation, causing a serious reduction in trabecular bone volume and thickness, causing defects in mineralization and causing reduced activity and proliferation of osteoblasts [10,11]. With the reduction of osteoblastic differentiation of bone marrow cells, adipogenesis occurs. In bone repair, alcohol decreases the synthesis of an ossifiable matrix, possibly due to the inhibition of cell proliferation and poor differentiation of mesenchymal cells in the repair tissue, generating deficient healing [10,12].

Bones are fundamental structures in supporting the body and are subject to bone loss originating from fractures, malformations or tumor resection [13]. This specialized tissue has a great capacity for spontaneous regeneration when the bone defect is not large [14]. When large losses occur, there is a need for intervention. With this, the use of biomaterials has become a great alternative in cases of bone regeneration [15]. A risk factor for fractures that can lead to the use of biomaterials is the chronic use of alcohol. After a fracture, alcoholics are more likely to have impaired healing and bone remodeling [16].

The expressive development and use of biomaterials for dental and medical clinic in the last decade has represented a powerful therapeutic tool in surgical activities, especially in the correction of bone defects [17]. Two biomaterials that are found on the market, Bio-Oss^®^ (Geistlich Pharma AG, Wolhusen, Switzerland) and OrthoGen^®^ (Baumer, Mogi Mirim, Brazil), are widely used and accessible for bone grafting. Bio-Oss^®^ inorganic bone matrix is composed of inorganic bovine bone, is widely used in various bone regeneration procedures in oral surgery [18,19], promotes bone formation and can be integrated into the bone modeling and remodeling process [20,21]. 

OrthoGen^®^ bone graft presents a typical structure of medullary bone, is essential for the successful deposition of osteoprogenitor cells on the graft and for resorption and the formation of new bone in place, and consists of an organic portion. Previous study by Galia et al. (2011) with OrthoGen^®^ demonstrated excellent biocompatibility, favoring the growth and development of new bone tissue [22].

Bio-Oss^®^ is extensively researched in the literature, with pre-clinical and clinical experiments cited by several studies, but we use this biomaterial, known worldwide in xenogenic grafts, comparing it to a product manufactured in Brazil, with a more accessible cost for its for use in medical and dental areas. This research is also justified by the fact that in the consulted literature, no comparison was found between these two biomaterials, especially when associated with the chronic use of alcohol present in alcoholic beverages.

Thus, we aimed to evaluate the biological behavior of two xenogenic scaffolds in alcohol-induced rats through histomorphometric and Picrosirius Red staining analysis of non-critical defects in the tibia of rats submitted or not to alcohol ingestion at 25% *v*/*v*.

## 2. Materials and Methods

### 2.1. Animals

Eighty adult male Wistar rats (*Rattus norvegicus*), 60 days old, weighing approximately 250 g, supplied by the Central Bioterium of Bauru School of Dentistry, University of São Paulo, Bauru, Brazil (FOB/USP), were used. The animals were kept in conventional cages containing four animals/box. The macroenvironment presented artificial timer-managed lighting, which controlled the 12/12-h light/dark cycle, with 220 lux brightness, 55% humidity, exhaust fan and air conditioning, maintaining an average temperature of 22 °C.

### 2.2. Ethical Aspects of Research

This study was conducted according to the guidelines of the Declaration of Helsinki and approved by the Institutional Ethics Committee on Animal Experimentation of the Bauru School of Dentistry, Bauru, Brazil (CEEPA/FOB), protocol code CEEPA—Proc. No 010/2014. Furthermore, this experimental study was carried out according to the ARRIVE guidelines and based on the principles of NC3Rs. Throughout the experimentation, the animals were monitored for expression of pain by observing whether the animal was apathetic, depressed, aggressive or overexcited, with such traits being variable in their usual behavior. Changes in walking, posture or facial expression were also observed, and the appearance, water consumption, food and clinical symptoms were investigated. There were no complications that needed to be reported, and there was no disease or sign that strongly motivated the removal of an animal (clinical outcome) [23].

### 2.3. Experimental Design

**Figure 1:**
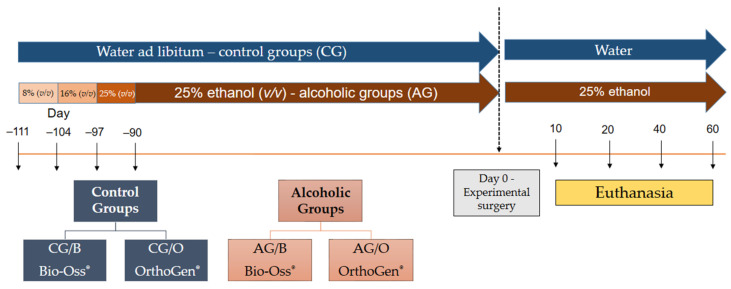
Experimental design (timeline): eighty adult male Wistar rats (*Rattus norvegicus*), aged 60 days, were divided into two groups: CG, control groups (*n* = 40), received only water with liquid diet; and AG, alcoholic groups (*n* = 40), received 25% ethanol solution (*v*/*v*) as a liquid diet after the adaptation period in progressive ethanol concentrations (8, 16 and 25% *v*/*v*). After 21 days of adaptation to alcohol, the animals retained 25% (*v*/*v*) until the surgical procedure (i.e., for 90 days). After making the bone defect filled with xenogenic biomaterials, four subgroups were pre-formatted according to the treatment: CG/B, water as liquid diet and surgical cavity in the tibia filled with Bio-Oss^®^; CG/O, water as a liquid diet and surgical cavity in the tibia filled with OrthoGen^®^; AG/B, 25% ethyl alcohol diluted in water and surgical cavity in the tibia filled with Bio-Oss^®^; and AG/O, 25% ethyl alcohol diluted in water and surgical cavity in the tibia filled with OrthoGen^®^. Euthanasia was performed 10, 20, 40 and 60 days after surgery, during which time the animals continued with the same liquid diet corresponding to the respective group.

The animals were randomly divided into four experimental groups containing 20 animals each, being: CG/B, water as liquid diet and surgical cavity in the tibia filled with Bio-Oss^®^; CG/O, water as a liquid diet and surgical cavity in the tibia filled with OrthoGen^®^; AG/B, 25% ethyl alcohol diluted in water and surgical cavity in the tibia filled with Bio-Oss^®^; and AG/O, 25% ethyl alcohol diluted in water and surgical cavity in the tibia filled with OrthoGen^®^ (Figure 1).

The animals received the same solid diet “ad libitum” throughout the experiment (Nuvilab^®^, Nuvital, Colombo, Brazil). The rats from Groups AG/B and AG/O were submitted to gradual adaptation to alcohol (absolute ethyl alcohol, Synth^®^, Diadema, Brazil), to induce chronic alcoholism and to prevent its death [24,25], being 7 days of an alcoholic diet at 8% (*v*/*v*), 7 days with alcohol at 16% (*v*/*v*) and 7 days at 25% (*v*/*v*), remaining in this last concentration until the end of the experiment (euthanasia).

### 2.4. Surgical Procedure

After 90 days of ethanol intake and induced dependence, all rats were weighed and subjected to intramuscular general anesthesia with intramuscular injection of tiletamine hydrochloride (125.0 mg), associated with zolazepam hydrochloride (125.0 mg), at a dosage of 50.0 mg/kg IM (Zoletil 50^®^, Virbac, São Paulo, Brazil), with strict monitoring of anesthesia mainly in alcoholic animals.

Trichotomy in the ventral region of the hind limb was followed by an incision with a scalpel blade No. 15, linear incision of 20 mm in length, in the craniocaudal direction, in the left pelvic limb, sectioning the skin and muscle fascia, for exposure and dilation of the muscle tissue surrounding the tibia. Obtaining a large working area over the tibia, with a spherical steel drill No. 6, in a low rotation micromotor, a cavity of approximately 3 mm in diameter was prepared, reaching the bone marrow in depth with abundant irrigation of 0.9% sodium chloride solution.

The animals in the experimental groups CG/B and AG/B received the filling of the cavity with the biomaterial Bio-Oss^®^. Animals of the CG/O and AG/O groups received the filling of the cavity with the OrthoGen^®^. The amount of biomaterial to fill the cavity was approximately 0.010 g of biomaterial for all groups, completely filling the perforation. After filling the cavities, the tissues in the surgical area were repositioned and sutured using 4-0 silk thread (Ethicon^®^, Johnson and Johnson, São Paulo, Brazil). After the surgical procedure, the animals were placed under incandescent light for complete anesthetic recovery and submitted to a single intramuscular injection of enrofloxacin 2.5 mg/kg (Flotril^®^; Schering-Plough SA, Rio de Janeiro, Brazil) and intramuscular injections of 0.06 mg/kg dipyrone (Analgex V^®^; Agener União, São Paulo, Brazil) for 3 days.

### 2.5. Biomaterials

Bio-Oss^®^ (Geistlich Pharma AG, Wolhusen, Switzerland) is an inorganic matrix of sterile cortical bovine bone composed of a structure and calcium-phosphorus ratio similar to hydroxyapatite of human bone (Ministry of Health, ANVISA—Brazil National Health Surveillance Agency, Registration No 806.969.30002). Characteristics of the demineralized bovine bone used in this study: graft type, demineralized bovine bone matrix, xenograft; granule size, granules of cancellous bone (0.25–1 mm); amount 2.0 g vial, 2.0 g vial; purification process, multi-phase (thermal deproteinization processes, temperature <900); sterilization, γ-irradiation; pore sizes, macropores (300–1500 µm) and micropores; porosity, 70–75% of the total volume; internal surface, area 100 m^2^/g and compressive strength, 35 MPa.

OrthoGen^®^ (Baumer, Mogi Mirim, Brazil) is composed of bovine bone marrow processed, dried and sterilized (ANVISA Registration No: 10345500098). The processing method allows for the maintenance of the natural characteristics of bone tissue, with a chemical composition of approximately 60–70% mineral phase (composed of hydroxyapatite), 20–30% organic phase and 8–9% water. In assessing the proximate chemical composition, we found the following contents: ash or ash = 60–70%, fat = < 1% total protein = 20–31%, calcium (Ca) = 20–25%, phosphorus (P) = 10–15%, and pH 6.8. In the determination of heavy metals, the following levels were found: As = < 3 ppm, Cd = < 5 ppm, Hg = < 3 ppm and Pb = < 30 ppm [22,26].

### 2.6. Collection of Specimens and Histological Procedures

Five animals from each group were euthanized using the afore mentioned anesthetic overdose at the respective periods of 10, 20, 40 and 60 days. The specimens were removed and fixed in 10% buffered formalin for 48 h and then demineralized in EDTA; a solution containing 4.13% Titriplex^®^ III (Merck KGaA, Darmstadt, Germany); and 0.44% sodium hydroxide, for a period of approximately 40 days. Then, the specimens were subjected to standard histological procedures and included in Histosec^®^ (Merck KGaA, Darmstadt, Germany). Histological sections were obtained with 5 µm thickness, prioritizing the defect centers for hematoxylin and eosin, Masson’s trichrome and Picrosirius Red staining.

### 2.7. Histomorphometric Evaluation

The histologic sections were analyzed in the Histology Laboratory of Bauru School of Dentistry, University of São Paulo (São Paulo, Brazil) by light microscopy (Olympus^®^ model BX50, Tokyo, Japan) at approximate magnifications of 4×, 10×, 40× and 100×. To establish a standard criterion for judgment, there was a training session with an experienced pathologist. For the two examiners trained for the analysis, the groups and experimental periods were blinded.

For histomorphological description of the bone defect area, the central region was considered with the aid of free-scale image capture system (DP Controller software^®^ 3.2.1.276 version, Olympus, Tokyo, Japan) to analyze tissue formation granulation, inflammatory infiltrate, formation of primary bone tissue, and bone maturation.

A virtual overall image of the defect (Masson’s trichrome, 10×) was generated to quantify the volume density (%) of newly formed bone tissue by AxioVision^®^ Rel. 4.8 Ink (Carl Zeiss MicroImaging GmbH, Jena, Germany). For determination of volumetric density (%), the equation Vvi = AAi = Ai/A × 100 was considered, considering Vvi (volume density), AAi (area density) and Ai (area filled with newly formed bone tissue).

Images from picrosirius-red stained sections were captured using a higher resolution digital camera Leica DFC 310FX (Leica^®^, Microsystems, Wetzlar, Germany) connected to a confocal laser microscope Leica DM IRBE and capture system LAS 4.0.0 (Leica^®^, Microsystems, Heerbrugg, Switzerland), allocated to the Integrated Research Center of the Bauru School of Dentistry. The quality of newly formed bone in the defects was evaluated by the orientation pattern and width of the collagen fibers, detected by the birefringence of polarization colors ranging from red-orange (primary disorganized bone tissue) to green-yellow (organized bone-lamellar bone tissue).

### 2.8. Statistical Evaluation of Data

Statistical analysis was performed in all groups and in all periods analyzed, observing the central area of the defect, ruptured cortical bone and bone tissue in formation. For the comparative analysis within each group, in all periods evaluated, the analysis of variance ANOVA test (ordinary one-way) with Tukey’s post-test (Tukey’s multiple comparisons test) was performed, with significance levels and considerations determined at *p* < 0.05. For the comparative analysis between the groups, in the same period evaluated, the unpaired *t*-test was performed, with a significance level of *p* < 0.05. The program used for statistical analysis was GraphPad^®^ Prism 8 (La Jolla, CA, USA).

## 3. Results and Discussion

### 3.1. Histomorphological Analysis

**Figure 2:**
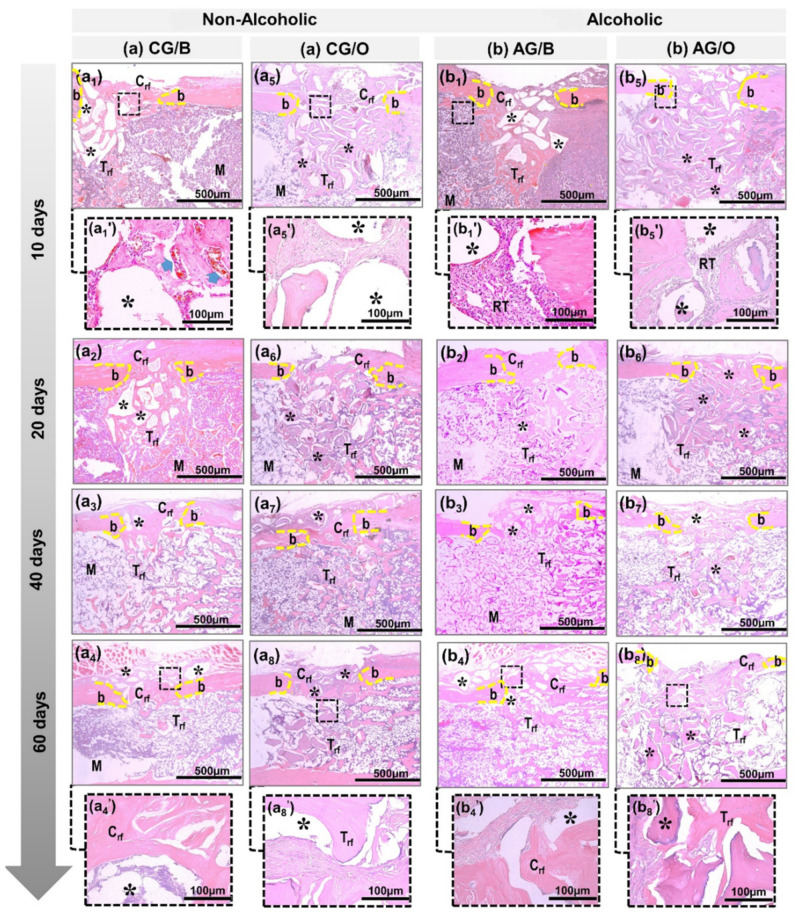
Details of the evolution of bone repair of defects in tibias created in non-alcoholic (CG/B and CG/O) animals and alcoholic (AG/B and AG/O) animals, and animals treated with Bio-Oss^®^ (**a1**–**a4**,**b1**–**b4**) or OrthoGen^®^ (**a5**–**a8**,**b5**–**b8**), at 10, 20, 40 and 60 days. Abbreviations: defect border (b); newly formed cortical bone (Crf); biomaterial particle (asterisk); newly formed trabecular bone (Trf); bone marrow (M); blood vessels (blue arrow); and tissue reaction (RT). Hematoxylin and eosin; original magnification 10×; bar = 500 µm and Insets, magnified images 40×; bar = 100 µm (**a1′**,**a4′**,**a5′**,**a8′**,**b1′**,**b4′**,**b5′**,**b8′**).

**Figure 3:**
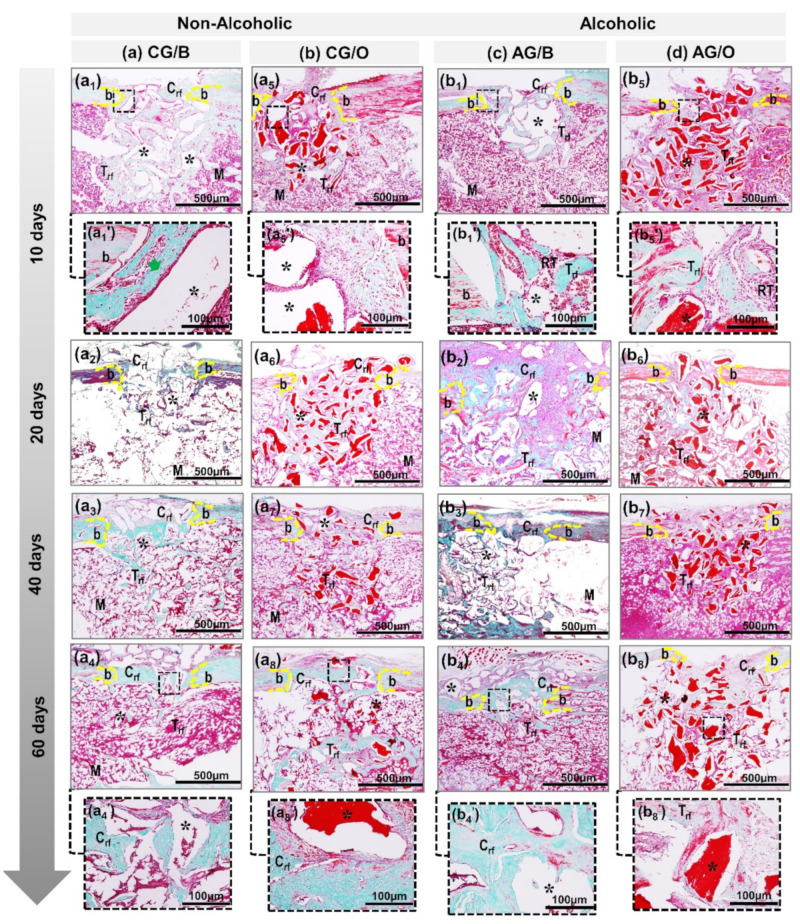
Details of the evolution of bone repair of defects in tibias created in non-alcoholic CG/B and CG/O animals and alcoholic AG/B and AG/O animals, and animals treated with Bio-Oss^®^ (**a1**–**a4**,**b1**–**b4**) or OrthoGen^®^ (**a5**–**a8**,**b5**–**b8**), at 10, 20, 40 and 60 days. Abbreviations: defect border (b); newly formed cortical bone (Crf); biomaterial particle (asterisk); newly formed trabecular bone (Trf); bone marrow (M); newly formed collagen fibers (green arrow); tissue reaction (RT). Masson trichrome; original magnification 10×; bar = 500 µm and insets, magnified images 40×; bar = 100 µm (**a1′**,**a4′**,**a5′**,**a8′**,**b1′**,**b4′**,**b5′**,**b8′**).

In all experimental groups, between 10 and 20 days (Figure 2 and Figure 3) there was a rupture of the cortical bone, with well-defined limits, and a slight bone formation, starting from the edges of the defect. The biomaterial particles transacted the cortical bone lesion, surrounded by loose connective tissue with the presence of vascular sprouts, being more evident in non-alcoholic animals treated with Bio-Oss^®^ xenograft (Figure 2(a1’)). In the medullary region, all analyzed groups presented clear particles of biomaterials, surrounded by disorganized collagen fibrils. However, the alcoholic animals, AG/B and AG/O, presented, both in the cortical and medullary regions, a reactive tissue with inflammatory infiltrate (Figure 2(b1’,b5’) and Figure 3(b1’,b5’)).

After 40 days, in non-alcoholic animals, CG/B and CG/O, in the area of the cortical lesion there was greater deposition of osteoid matrix, already in the process of bone maturation, with a reduction in the particles of biomaterials. In the medullary area, there was the presence of medullary connective tissue, with newly formed bone trabeculae surrounding the biomaterial particles (Figure 2 and Figure 3 non-alcoholic). In alcoholic animals, AG/BA and AG/O, the inflammatory process was in the resolution phase, with sparse reactive cell alterations in the spinal tissue (Figure 2 and Figure 3 alcoholic).

In 60 days, the central area of the cortical bone still presented, in all groups, particles of biomaterials, surrounded by newly formed compact tissue, without complete closure of the lesion, except in non-alcoholic animals treated with xenografts, CG/B, which interconnected the edges of the defect by lamellar bone tissue (Figure 2(a4) and Figure 3(a4)). In the medullary region, compact bone trabeculae and the presence of biomaterial particles were observed, more expressive in the groups treated with the allograft, CG/O and AG/O (Figure 2(a8,b8) and Figure 3(a8,b8).

Bio-Oss^®^ acted positively in animals that received water as a liquid diet (CG/B group), serving as a scaffold for the proliferation of bone cells in the remodeling process, in agreement with the results of previous studies, which demonstrated the formation of new bone through the proliferation of osteoblastic cells [19,27], in addition to presenting beneficial properties of osteoconductibility, predictability in treatment and good prognosis [28,29]. In animals that received alcohol as a liquid diet (AG/B), Bio-Oss^®^ also contributed to bone neoformation but with a greater delay compared to animals with liquid diet water. Microscopically, the presence of inflammatory cells, connective tissue and bone tissue in the beginning of remodeling was observed. Alcohol interfered negatively in bone neoformation, presenting a greater delay in the entire process. The literature agrees with the results obtained as it points out that the chronic use of alcohol inhibits and delays bone formation [10,24,30,31,32].

The xenogenic biomaterial OrthoGen^®^, in the groups that received the alcoholic or non-alcoholic diet (CG/O and AG/O), also contributed to the bone neoformation around the particles but presented a lower volume density and was slower than Bio-Oss^®^. Previous studies showed that OrthoGen^®^ has the potential to form new bone tissue due to its three-dimensional structure, acting as a scaffold, which is essential for osteoblastic cells, in addition to being biocompatible and low-cost [22,33]. Alcohol interfered negatively, delaying the repair process, which is in agreement with previous studies, pointing out that chronic alcohol consumption inhibits mechanisms responsible for cell proliferation, which act directly on bone formation and remodeling [34,35,36].

### 3.2. Collagen Fiber Birefringence Analysis

**Figure 4:**
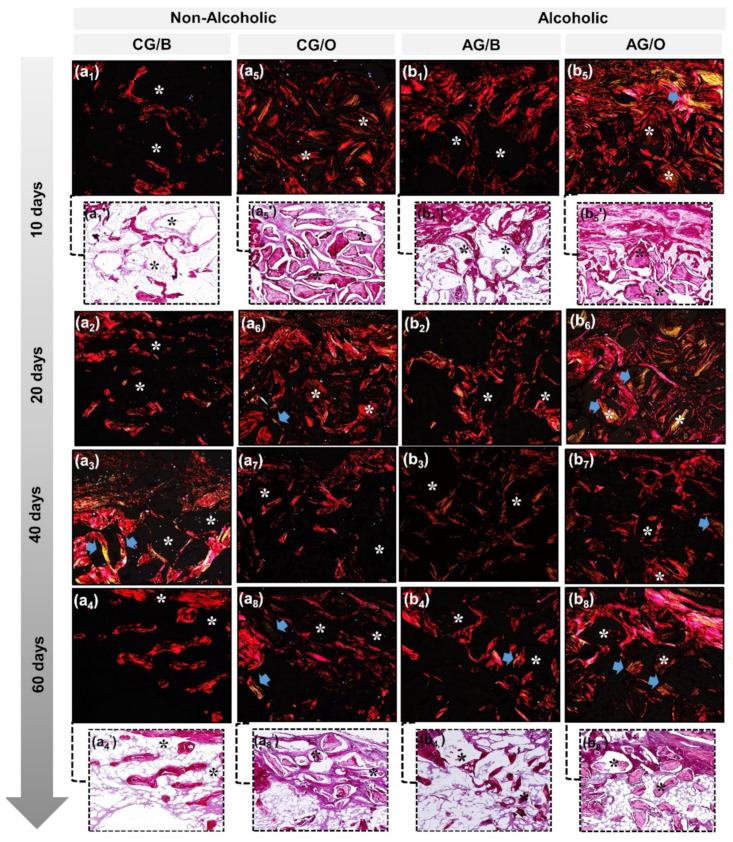
Birefringence analysis of newly formed collagen matrix by polarized microscopy exemplified in non-alcoholic (CG/B and CG/O) animals and alcoholic (AG/B and AG/O) animals, treated with Bio-Oss^®^ (**a1**–**a4**,**b1**–**b4**) or OrthoGen^®^ (**a5**–**a8**,**b5**–**b8**) at 10, 20, 40 and 60 days. New bone formation with red-orange birefringence, referring to the osteoid matrix, gradually changing to greenish-yellow (blue arrow) at the end of the experimental period, xenograft, Bio-Oss^®^, identified by a dark background field (asterisk) and allograft parts dark field and parts by birefringence of collagen fibers corresponding to the organic portion of the biomaterial. Staining with Picrosirius Red, original magnification 10×. Magnified images 40× (**a1′**,**a4′**,**a5′**,**a8′**,**b1′**,**b4′**,**b5′**,**b8′**).

In the initial periods, 10 and 20 days, in all non-alcoholic CG/B and CG/O and alcoholic AG/B and AG/O animals, a red-orange birefringence color was observed, corresponding to disorganized collagen fibrils, involving the biomaterial particles. These particles were evidenced in a dark field that remained for up to 60 days. However, the defects filled with the allograft, OrthoGen^®^, CG/O and AG/O, in addition to the dark field referring to the inorganic part of the biomaterial, presented thick collagen fibers with greenish-yellow birefringence, concerning the organic part of the same (Figure 4(a5,b5)).

From 40 days until the end of the analyzed period, in all experimental groups there was a slight transition from birefringence to greenish-yellow, consistent with lamellar bone formation and bone maturation process, mainly surrounding the biomaterial particles (Figure 4(a3,a4,a7,a8,b3,b4,b7,b8).

This birefringence transition observed in the histochemical analysis of collagen fibers by Picrosirius Red, in which all groups in periods of 10 and 20 days showed red-orange birefringence, and from 40 days onwards greenish-yellow, demonstrates the characteristic transition from the formation of thin and disorganized collagen fibers initially to more organized and thicker later [37]. The biomaterial particles exhibited birefringence close to the newly formed bone tissue, preventing the measurement of specific fibers from bone repair.

### 3.3. Histomorphometric and Statistical Analysis

**Figure 5:**
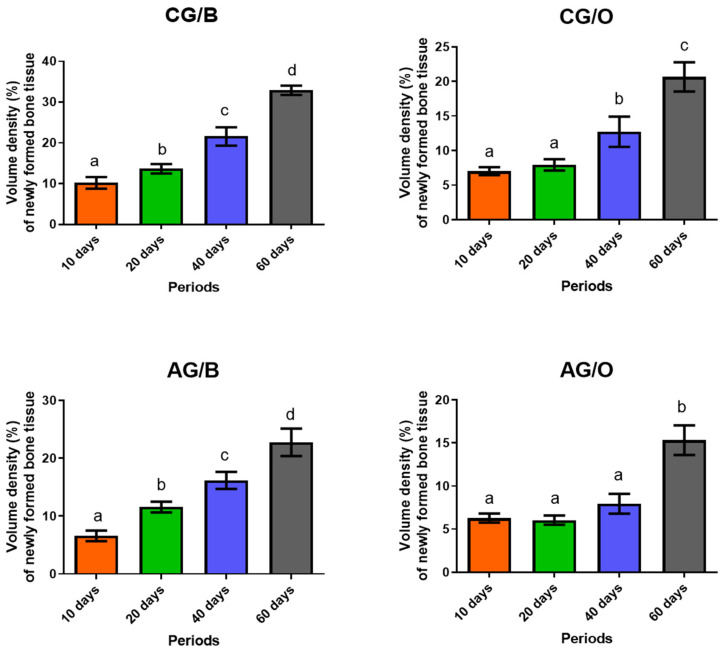
Quantification of the percentage of new bone tissue (mean ± SD) of the experimental groups in the analyzed periods. Different lowercase letters (line) indicate a significant difference between the analyzed periods. (a ≠ b ≠ c ≠ d). Significant differences (*p* < 0.05). Non-alcoholic groups (CG/B and CG/O) alcoholic groups (AG/B and AG/O), and animals treated with Bio-Oss^®^ (CG/B and AG/B) or OrthoGen^®^ (CG/O and AG/O), at 10, 20, 40 and 60 days.

In the quantitative analysis of the percentage of new bone formed, when the experimental groups were studied individually, comparing the four experimental periods (10, 20, 40 and 60 days), it was observed that in Group CG/B (water + Bio-Oss^®^) there was a significant difference in all periods, with an increase in bone formation. In CG/O (water + OrthoGen^®^), there was no significant difference between the periods of 10 and 20 days. In the periods of 40 and 60 days, there was a gradual bone development, with a significant difference between them, similarly to the previous periods (10 and 20 days) (Table 1 and Figure 5).

In the groups with a liquid alcoholic diet, in AG/B (ethanol + Bio-Oss^®^), it was observed that in all periods, when comparing the percentage of bone tissue in formation, there was an increasing average of bone development between the groups, with significant difference between all periods. In AG/O (ethanol + OrthoGen^®^), there was a significant difference only in the period of 60 days compared to all previous periods analyzed (10, 20 and 40 days) (Table 1 and Figure 5).

Comparison of the results of the percentage of new bone formed between the experimental groups, in the analyzed periods, was also carried out. Initially, animals were given the same liquid diet and different graft biomaterials.

**Figure 6:**
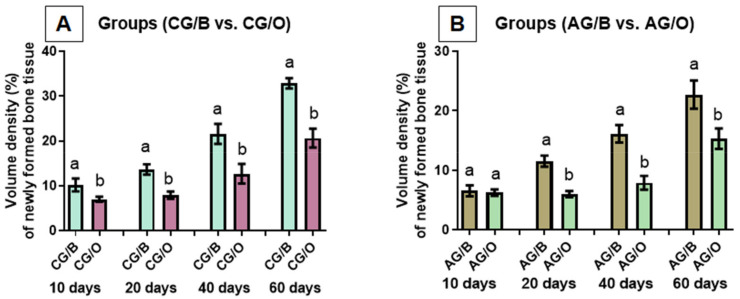
Comparison of the percentage of new bone tissue (mean ± SD) of the groups with the same liquid diet of water (**A**), non-alcoholic or (**B**) alcoholic (25% *v/v* ethanol), with different graft materials. Different lowercase letters indicate a significant difference between the analyzed groups (a ≠ b). Significant differences (*p* < 0.05). Non-alcoholic groups (CG/B and CG/O) alcoholic groups (AG/B and AG/O), and animals treated with Bio-Oss^®^ (CG/B and AG/B) or OrthoGen^®^ (CG/O and AG/O), at 10, 20, 40 and 60 days.

In the groups that received water as a liquid diet (non-alcoholic, CG/B and CG/O) but with different biomaterials, there was a significant difference between the groups in all periods. The CG/B group (Bio-Oss^®^) presented a higher percentage of bone formation compared to the CG/O group (OrthoGen^®^), in all periods (Figure 6A).

When groups AG/B and AG/O were compared, with the same liquid diet (ethanol 25% *v*/*v*), with different biomaterials, it was observed that within 10 days there was no significant difference. In the periods of 20, 40 and 60 days, there was a significant difference between the groups, where the group with the biomaterial Bio-Oss^®^ (AG/B) presented a higher percentage of bone formation when compared to the OrthoGen^®^ group (AG/O) (Figure 6B).

**Figure 7:**
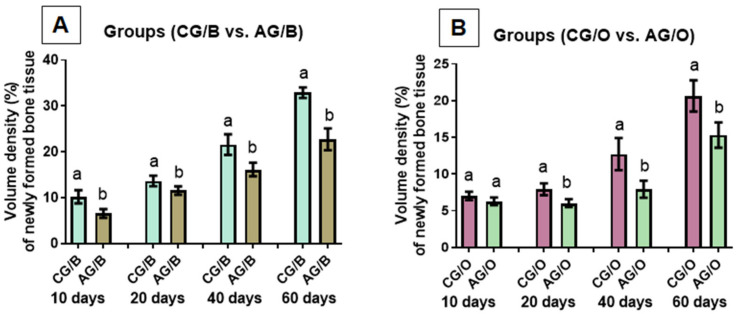
Comparison of the percentage of new bone tissue (mean ± SD) of groups with different liquid diets, alcoholic (AG/B and AG/O) or non-alcoholic (CG/B and CG/O), with the same graft biomaterial (**A**) Bio-Oss^®^ or (**B**) OrthoGen^®^, at 10, 20, 40 and 60 days. Different lowercase letters indicate a significant difference between the analyzed groups (a ≠ b). Significant differences (*p* < 0.05).

Comparing the percentage of new bone in the groups that used the same graft material but with different liquid diets, it was observed that when using Bio-Oss^®^ (CG/B vs. AG/B), a significant difference was observed in all periods evaluated (10, 20, 40 and 60 days), and the group that received water as a liquid diet (CG/B) had higher means (mean ± SD) (Figure 7A).

Using the biomaterial OrthoGen^®^, within 10 days there was no significant difference between the groups evaluated (CG/O vs. AG/O). In the periods of 20, 40 and 60 days, there was a significant difference in the percentage of bone formation, and the group that received the liquid water-based diet (CG/O) obtained greater bone formation (Figure 7B).

The quantitative results showed that the groups that received the alcoholic diet (AG/B and AG/O) presented a delay in bone formation in the cortical region and in the spongy bone tissue where the biomaterial was implanted. The effects of ethanol suppress bone cortical and spongy tissue in the bone remodeling period [38,39]. The chronic user of alcoholic beverages can present a low bone mineral density, being totally linked to the risk of fractures, osteoporosis and delay in bone remodeling [40]. In the two biomaterials tested, Bio-Oss^®^ showed greater bone formation and has been the first choice in bone grafting procedures of xenogenous origin, as the particles of the inorganic graft are progressively reabsorbed and then replaced by new bone, with structural characteristics similar to human bone [41]. It performed well in the mineralization, and its particle was incorporated into the newly formed bone [42].

OrthoGen^®^, due to the results obtained, can also be used as a bone substitute even though it does not reach the results of Bio-Oss^®^ as it proved to be biocompatible and a scaffold for the repair process, in agreement with previous research, in which the biomaterial proved to be capable to be incorporated and lead to bone neoformation [43]. This biomaterial has good results when analyzed in the long term, being of safe and effective use in the restoration of defects [22,44].

Regardless of the diet and the biomaterial used, some complementary therapies have the ability to favor and increase the volume of new bone formed, reducing postoperative recovery time, reducing edema and pain, such as photobiomodulation with the use of low-level laser [45,46,47,48,49]. Photobiomodulation was also able to increase the expression of myogenic and vascular growth factors and stimulate skeletal muscle regeneration in rats with chronic alcohol intake [50].

## 4. Conclusions

This study aimed to evaluate the bone defects repair process using a Brazilian-made mixed bovine bone matrix (OrthoGen^®^) in relation to a biomaterial of worldwide use (Bio-Oss^®^), in rats submitted to the ingestion of diet by water or 25% *v/v* ethanol. The formation of new bone occurred in defects that received the two biomaterials, in all periods evaluated (10, 20, 40 and 60 days), but Bio-Oss^®^ was superior in the results evaluated by histomorphology, histomorphometry and collagen birefringence, regardless of the type of liquid diet. The alcoholic diet interfered negatively in the percentage of new bone formed, and Bio-Oss^®^ mainly minimized this exposure.

Despite all the analysis carried out in this study, we can consider as a limitation the failure to perform an immunohistochemical analysis, which would favor the understanding of bone formation. Furthermore, it could be associated with metabolic dysfunction with alcohol consumption and its influence on the bone repair process [51,52]. There is a need for further studies as the number of patients who receive orthopedic and dental procedures, and who chronically use alcoholic beverages, has increased considerably. It is increasingly necessary to clarify the risks and harms of alcoholic beverages, especially for patients who are recovering from bone loss, whether due to fracture or trauma.

## Figures and Tables

**Table 1:** Quantification of the percentage of new bone tissue (mean ± standard deviation (SD)) of the experimental groups in the analyzed periods (10, 20, 40 and 60 days).

Groups	10 Days	20 Days	40 Days	60 Days
CG/B	10.22 ± 1.42 a	13.66 ± 1.15 b	21.60 ± 2.26 c	32.9 ± 1.15 d
CG/O	7.02 ± 0.56 a	7.92 ± 0.81 a	12.72 ± 2.18 b	20.66 ± 2.12 c
AG/B	6.58 ± 0.92 a	11.57 ± 0.92 b	16.16 ± 1.48 c	22.74 ± 1.15 d
AG/O	6.28 ± 0.52 a	6.04 ± 0.53 a	7.94 ± 1.15 a	15.32 ± 1.71 b

Different lowercase letters (line) indicate a significant difference between the analyzed periods. (a ≠ b ≠ c ≠ d). Significant differences (*p* < 0.05). Non-alcoholic groups (CG/B and CG/O), alcoholic groups, (AG/B and AG/O), and animals treated with Bio-Oss^®^ (CG/B and AG/B) or OrthoGen^®^ (CG/O and AG/O), at 10, 20, 40 and 60 days.

## Data Availability

The data presented in this study are available on request from the corresponding author. The data are not publicly available due to they are part of a master’s dissertation not yet deposited in a public repository.

## Sample Availability

Samples of the Bio-Oss^®^ and OrthoGen^®^ matrices are available from the authors.
